# Metastatic Unfunctional Pancreatic Neuroendocrine Tumor in Lynch Syndrome

**DOI:** 10.1002/ccr3.73036

**Published:** 2026-06-29

**Authors:** Fateme Salemi, Zahra Sadin, Atefe Barzegari, Seyed Mohammad Reza Mortazavizadeh, Massih Bahar, Motahare Amiri

**Affiliations:** ^1^ Hematology, Oncology and Stem Cell Transplantation Research Center, Research Institute for Oncology, Hematology and Cell Therapy Tehran University of Medical Sciences Tehran Iran; ^2^ Department of Stem Cells and Developmental Biology Cell Science Research Center, Royan Institute for Stem Cell Biology and Technology, ACECR Tehran Iran; ^3^ Department of Hematology and Oncology Islamic Azad University, Yazd Branch Yazd Iran; ^4^ Department of Clinical Biochemistry Tehran University of Medical Sciences, Faculty of Medicine Tehran Iran; ^5^ Department of Operative Dentistry, School of Dentistry Shahid Sadoughi University of Medical Sciences Yazd Iran

**Keywords:** dMMR, Lynch syndrome, MutL homolog‐ 1, pancreatic neuroendocrine tumor

## Abstract

Lynch syndrome (LS), a well‐known cancer risk syndrome, is caused by deleterious germline mutations in the mismatch repair genes. LS predispose patients to various types of cancers including colon adenocarcinoma. We discuss the case of a woman with LS who also developed a non‐functioning pancreatic neuroendocrine tumor (P‐NET) following primary colon adenocarcinoma. Multiple liver lesions were discovered 6 months following surgical excision the pancreatic mass and were later determined to be a neuroendocrine tumor. Liver lesions shrank after treatment with octreotide and Lutetium‐177 vipivotide tetraxetan. She is in remission and takes positron emission tomography scans every 6 months for monitoring. This case highlights the importance of LS genetic testing, the function of microsatellite instability (MSI) as a screening marker, and the need for additional study on the relationship between LS and P‐NETs, particularly through advanced molecular testing to confirm lesion relationships. The role of microsatellite instability (MSI) as a screening marker, and personalized treatment approaches like immunotherapy for dMMR tumors. Understanding these correlations may assist in early discovery, surveillance, and customized treatment for patients dealing with LS‐associated malignancies.

## Key Clinical Message

Lynch syndrome can present with uncommon tumors in the colon and uterus. We report a young woman with a rare pancreatic neuroendocrine tumor 7 years after colon cancer. It shows unusual molecular patterns between primary and metastatic sites. Our targeted therapy controlled her disease for 3 years. It highlights the importance of cancer surveillance in these patients.

## 1 Introduction

Lynch syndrome (LS), which was known as hereditary non‐polyposis colorectal cancer (HNPCC), is present in 1 in 279 people around the world. It is the most common hereditary cause of colorectal cancer (CRC) and cause about 2%–4% of all CRC cases [1]. It results from a germline pathogenic (P) or likely pathogenic (LP) variant in one of the DNA mismatch repair (MMR) genes, MLH1, MSH2, MSH6, or PMS2, or in the EPCAM gene, these deletions cause MSH2 inactivation [2, 3]. Data from hereditary cancer registries show that specific MMR gene variants are associated with different cancer risks. The most malignancies that are associated to LS are cancers of the colon, rectum, endometrium, ovary, stomach, renal pelvis, ureter, bladder, small intestine, pancreas, skin (particularly sebaceous neoplasms), biliary tract, and brain [4]. Besides, multiple reports from hereditary cancer registries and institutional series describe rare or atypical tumor types that occurre in the context of LS [5].

LS‐associated malignancies are characterized by deficient DNA mismatch repair (dMMR), which results in a high level of microsatellite instability (MSI‐H) and an elevated tumor mutational burden (TMB). Molecular assays assessing MSI status and MMR protein expression are routinely used to detect MSI‐H/dMMR phenotypes [6]. MSI‐H tumors, in the context of LS or sporadically, show distinct biological and clinical behavior compared with microsatellite‐stable (MSS) tumors, it makes them appropriate candidates for immunotherapy and other emerging targeted strategies [7]. In contrast to LS‐related colorectal cancers, sporadic MSI‐H CRCs are typically associated with somatic BRAF V600E mutations or MLH1 promoter hypermethylation [8]. These molecular features are clinically useful for distinguishing germline‐associated dMMR from sporadic dMMR colorectal cancers [8].

Pancreatic neuroendocrine tumors (P‐NETs) are a subtype of gastroenteropancreatic neuroendocrine neoplasms (GEPNENs) that arise from the‐ hormonesecreting cells of the pancreatic neuroendocrin‐e system and represent the second most common form of pancreatic neoplasm [9]. Although most P‐NETs occur sporadically, a subset develops in association with hereditary cancer predisposition syndromes such as multiple endocrine neoplasia type 1 (MEN1), von Hippel–Lindau (VHL) syndrome, tuberous sclerosis complex (TSC), and neurofibromatosis type 1 (NF1) [10]. Neuroendocrine tumors are uncommon in Lynch syndrome, and the combination of Lynch syndrome with a pancreatic neuroendocrine tumor (P‐NET) has been only sporadically reported [9, 11]; however, a definitive causal association is still vague. We report a case of a young woman with MLH1‐related LS who presented with primary colon adenocarcinoma and a non‐functional metastatic P‐NET involving the liver.

## 2 Case History/Examination, Differential Diagnosis, Investigations, and Treatment

In April 2014, a 29‐year‐old female with a strong family history of CRC was presented with abdominal discomfort, iron deficiency anemia and positive occult blood in stool exam. Colonoscopy showed a mass in the ascending colon. She had a total colectomy, proximal proctectomy, ileorectal anastomosis, and lymph node dissection. The gross pathology of the resected lesion revealed an annular tumor with a maximum diameter of 6 cm that was obstructing the lumen. Histopathology showed a dMMR pT3N0 low‐grade mucin‐producing ascending colon adenocarcinoma.

Following surgical resection, the patient received a 6‐month course of adjuvant chemotherapy with oxaliplatin and capecitabine (XELOX regimen). This decision was made based on the patient's young age, extensive family history, and pericolic fat involvement, before dMMR prognostic implications were fully integrated into practice. Current guidelines support observation alone for dMMR stage II colon cancer.

Although current NCCN and ESMO guidelines generally do not recommend adjuvant chemotherapy for dMMR/MSI‐H stage II colon cancer due to excellent prognosis and lack of proven benefit from fluoropyrimidine‐based regimens, this treatment decision was made in 2014 when the prognostic implications of dMMR status in stage II disease were not yet fully integrated into routine clinical practice. The decision was influenced by the patient's young age, extensive family history meeting Amsterdam criteria, and the potential for hereditary cancer syndrome, leading to a more aggressive approach at that time. Current evidence supports observation alone for dMMR stage II colon cancer without high‐risk features.

Immunohistochemistry (IHC) revealed loss of expression of MLH1 and PMS2 proteins. PCR using the NCI‐recommended Bethesda MSI panel showed MSI‐H. To distinguish between somatic and germline dMMR, MLH1 promoter methylation analysis and BRAF V600E mutation testing were performed on DNA extracted from formalin‐fixed paraffin‐embedded (FFPE) tissue by pyrosequencing (QIAGEN, Germany). MLH1 promoter analysis did not show hypermethylation, and BRAF V600 genotyping was wild, indicating germline or double somatic dMMR.

**FIGURE 1:**
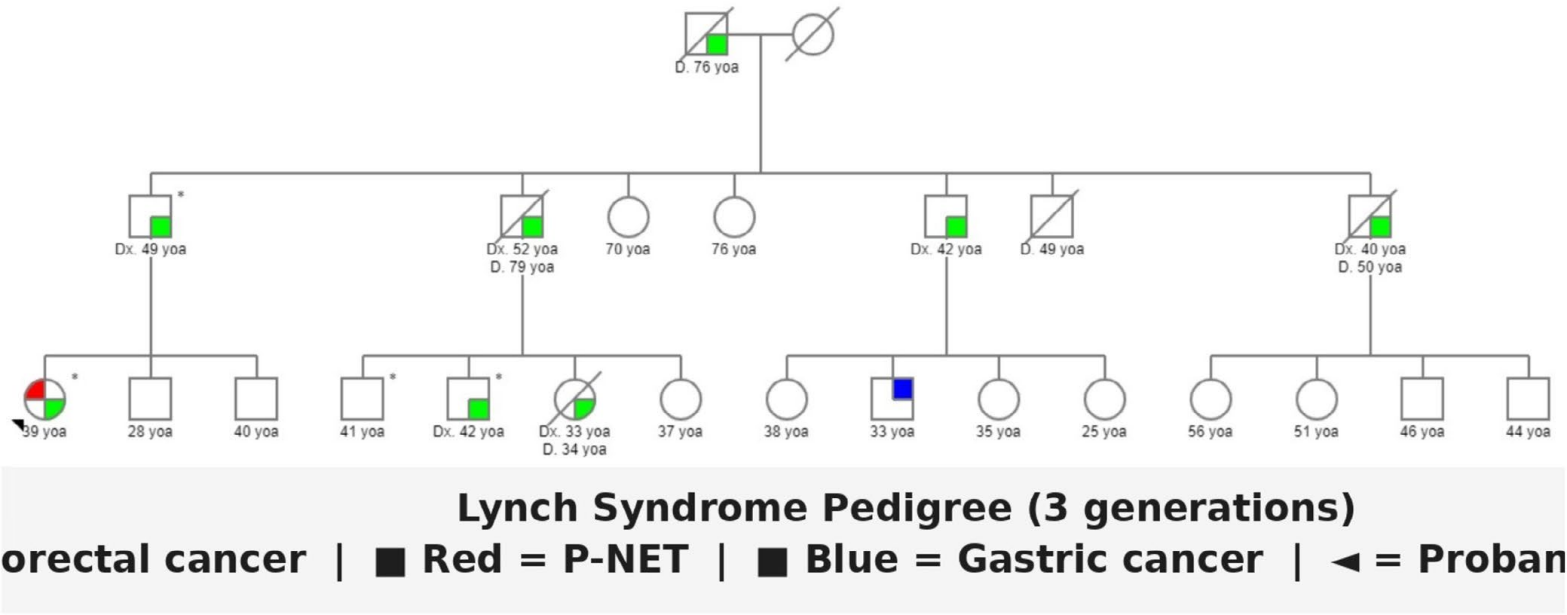
Familial pedigree of the Lynch syndrome (LS) index case and her relatives. Three generation pedigree of the presented case: Green quadrant signifies colorectal cancer; red signifies PNET, and blue signifies gastric cancer. Our case (the proband) is designated by an arrowhead.

Germline genetic panel testing revealed a c.131C>A (p.Ser44Tyr) variant in the MLH1 gene. At the time of our germline testing in 2017, p.Ser44Tyr was classified as a variant of unknown significance (VUS). The patient's family history met both Bethesda and Amsterdam criteria [12] (Figure 1). Combined clinical, histopathological, and molecular evidence supported the diagnosis of Lynch syndrome (LS); therefore, the patient was referred to the FamCan Institute for enrollment in an active surveillance program. Pathogenicity evidence for this variant was submitted to the Leiden Open Variation Database (LOVD). Subsequently, in 2018, p.Ser44Tyr was reclassified as pathogenic, confirming the diagnosis of LS.

After 7 years of living symptom‐free, at the age of 36, in August 2021 the patient presented with pruritus and elevated alkaline phosphatase (ALP: 1091). Abdominopelvic ultrasound showed dilation of the common bile duct (CBD) (13 mm). An echogenic area measuring 11 mm at the distal part of the CBD, and an echogenic lesion (19 × 25) at the pancreatic head were also detected. On spiral abdominal computed tomography (CT) scan with and without contrast a low‐density pancreatic head mass lesion measuring 23 × 24 × 27 mm was detected which caused pressure effect on the dilated CBD (12 mm), gall bladder, and intrahepatic bile duct. The findings were highly suggestive of obstructive jaundice due to neoplasm of the head of the pancreas without superior mesenteric artery (SMA) invasion.

**FIGURE 2:**
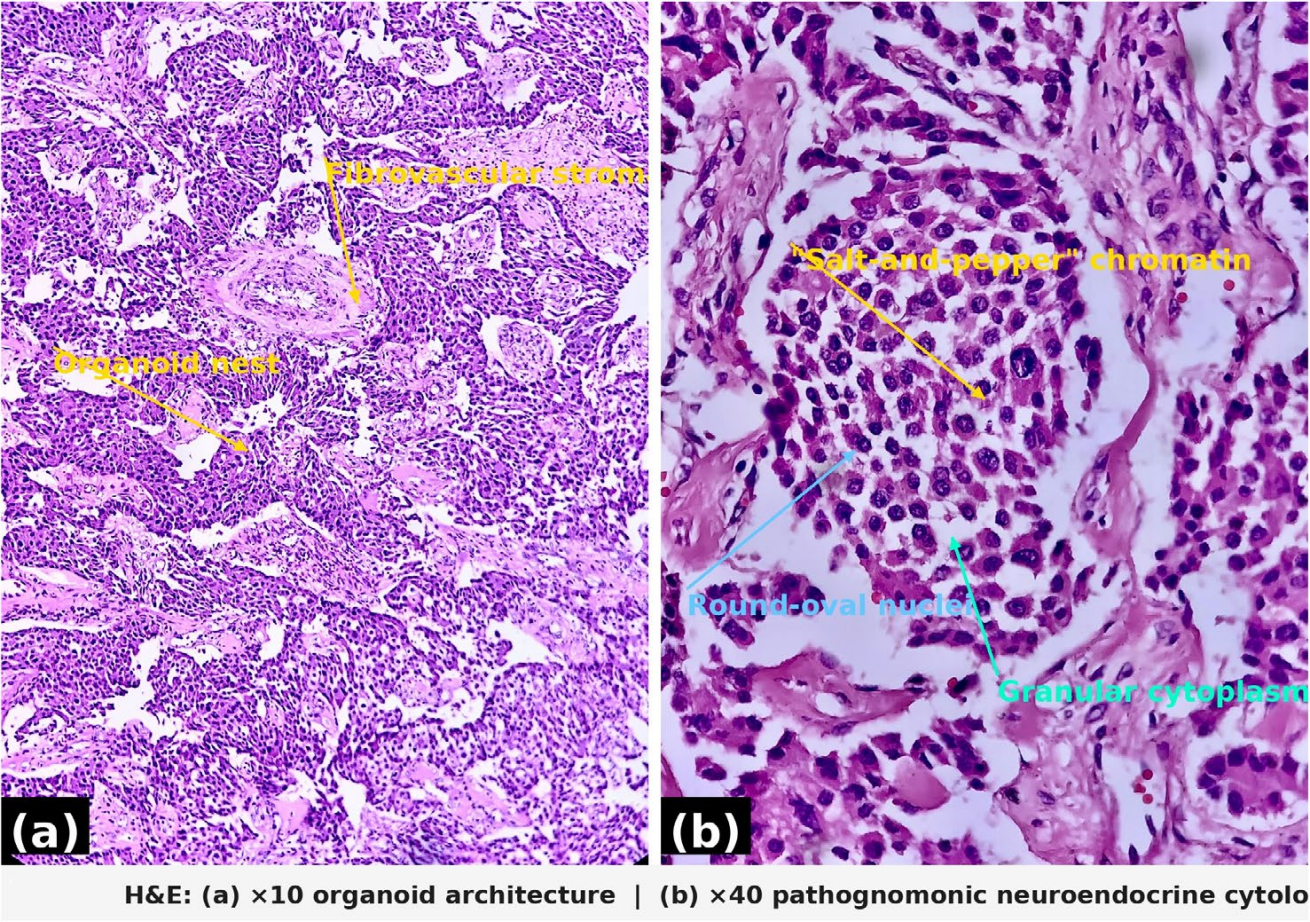
Hematoxylin and Eosin (H&E) staining of pancreatic sample 10× (a) and 40× (b) magnification.

Endoscopic ultrasound (EUS)‐guided fine‐needle aspiration (FNA) of the pancreatic mass and liver hilar lymph node demonstrated a well‐differentiated neuroendocrine neoplasm (Figure 2). Immunohistochemical staining of the biopsy specimen showed diffuse synaptophysin positivity, chromogranin A positivity in 5% of tumor cells, and a Ki‐67 proliferation index of 5%, consistent with a Grade 2 (G2) pancreatic neuroendocrine tumor (P‐NET) according to WHO 2019 classification.

**FIGURE 3:**
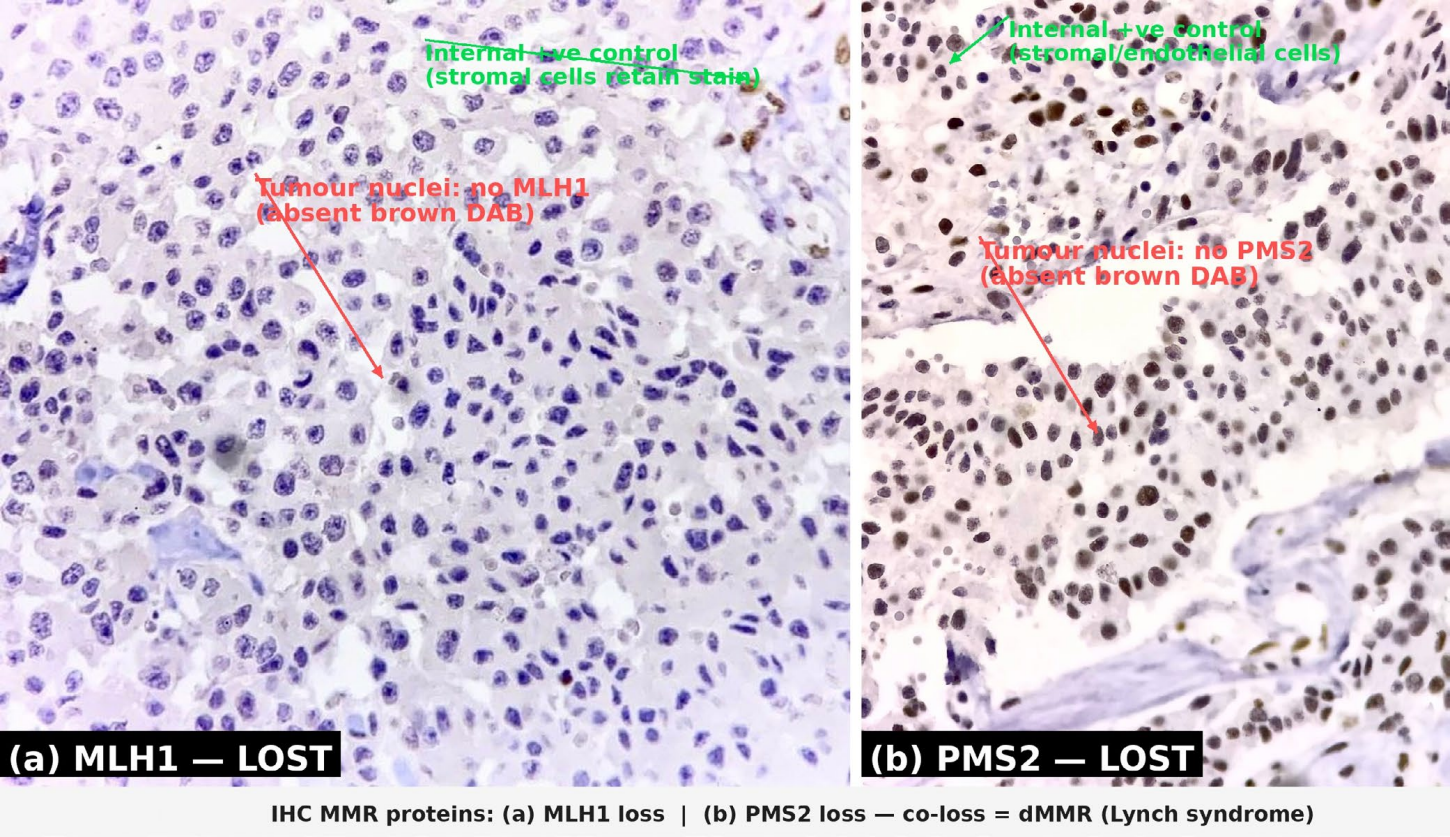
Immunohistochemistry (IHC) study of pancreatic tumor was positive for MLH1 (a) and MSH‐2 (b) mutations.

Pathological examination of the resected specimen revealed a 2.7‐cm well‐differentiated neuroendocrine tumor confined to the pancreas with peripancreatic soft tissue extension (pT2N0). All 28 regional lymph nodes were negative for malignancy. IHC analysis confirmed synaptophysin, chromogranin A, and INSM‐1 positivity in tumor cells with a Ki‐67 index of 4%, confirming G2 P‐NET. Notably, IHC also demonstrated loss of MLH1 and PMS2 protein expression in P‐NET cells, which is an unusual finding in neuroendocrine tumors. The dMMR result was confirmed by a second independent laboratory, establishing this as a G2 P‐NET arising in the context of LS (Figure 3). This represents one of the rare documented cases of Lynch syndrome‐associated pancreatic neuroendocrine tumors.

The patient underwent neuroendocrine tumor follow up with positron emission tomography scan (PET scan) every 6 months for follow up. Following resection of the localized P‐NET, the patient entered a structured surveillance program based on NANETS (North American Neuroendocrine Tumor Society) and ENETS (European Neuroendocrine Tumor Society) guidelines for resected P‐NETs. According to these guidelines for a resected Grade 2 non‐functional P‐NET, the patient underwent contrast‐enhanced multiphasic MRI as the primary imaging modality, performed every 6 month until the first 2 years post‐resection. Somatostatin receptor‐based imaging, such as Ga‐68 DOTATATE PET‐CT, was reserved for suspected recurrence with equivocal findings on Sonography, CT, or MRI. Initial postoperative ultrasound findings suggesting subtle hepatic parenchymal changes, Ga‐68 DOTATATE PET‐CT was performed at 6 months post‐resection to definitively assess for somatostatin receptor (SSTR)‐expressing disease that might not be evident on conventional imaging. This approach, however is not routine surveillance per guidelines, was performed given the high‐risk clinical context.

**FIGURE 4:**
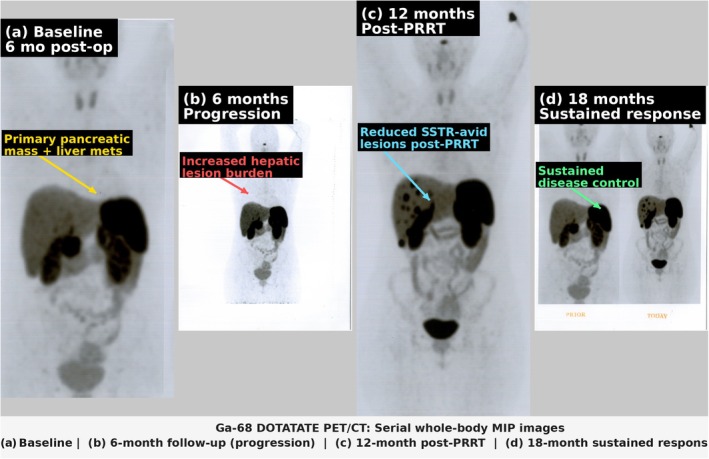
Serial follow‐up Ga‐68 DOTATATE PET CT scans showing the liver lesions and their response to treatment; (a) At the time of diagnosis the pancreatic mass, (b) 6 months later, (c) 12 months later, d: 18 months later.

Ga‐68 DOTATATE PET scan showed multiple liver lobes lesions and mesenteric soft tissue enhancements highly suggestive of metastasis (Figure 4a,b).

Ultrasound‐guided core needle biopsy of the hepatic lesion revealed well‐differentiated metastatic neuroendocrine tumor, Grade 1 (Ki‐67 1%). Immunohistochemical staining showed Positive Cytokeratin (CK), synaptophysin, INSM‐1 and Negative Chromogranin A Among MMR proteins, Surprisingly, MLH1 and PMS2 were intact (retained expression). This discordant MMR protein expression pattern between the pancreatic tumor (MLH1/PMS2 loss) and hepatic lesion (MLH1/PMS2 intact) prompted further molecular investigation.

To clarify the relationship between the pancreatic and liver lesions, next‐generation sequencing (NGS) was performed on Formalin‐Fixed Paraffin‐Embedded (FFPE) tissue from both sites using a comprehensive cancer panel. Analysis revealed Shared germline MLH1 c.131C>A (p.Ser44Tyr), pathogenic variant in both lesions, which confirmed clonal relationship and discordant somatic mutational profiles with distinct additional alterations in each tumor. There was no evidence of MLH1 reversion mutations in the metastatic lesion.

We used SSA (octreotide LAR 30 mg every 28 days) and continued for 3 months with radiographic assessment. Besides, we perform close monitoring with contrast‐enhanced CT at 3‐month intervals per guidelines.

At 3‐month follow‐up imaging (June 2022), disease progression was documented: hepatic metastases increased in size and number (RECIST 1.1 criteria: 35% increase in sum of target lesions). Also, new hepatic lesions appeared in previously uninvolved segments. Therefore, given documented progression on SSA and strongly SSTR‐positive disease, the patient was transitioned to peptide receptor radionuclide therapy (PRRT) with Lu‐177 DOTATATE (Lutathera:** 7.4 GBq (200 mCi) intravenously every 8 weeks for 4 cycles) in July 2022, octreotide LAR administration (30 mg every 28 days) was continued between PRRT cycles (discontinued 24 h before each infusion). Fortunately, the patient is performing well and symptom‐free, with sustained disease control since today.

The clinical protocol was approved by the institutional ethics review board of Yazd Azad University of Medical Sciences. Written informed consent was obtained from the patient prior to initiation of treatment and for publication of this case report.

## 3 Conclusion

This case highlights a rare Lynch syndrome (LS)‐associated pancreatic neuroendocrine tumor (P‐NET) with hepatic metastases, which is characterized by MMR heterogeneity (MLH1/PMS2 loss in G2 primary, intact in G1 metastases). It has been confirmed by NGS as clonally related. LS‐P‐NETs, primarily linked to MLH1, are uncommon, so it warrants broader surveillance beyond colorectal and endometrial MSI screening. Sustained 3+ year disease control with somatostatin analogs and PRRT, which is prioritized over immune checkpoint inhibitors due to limited NET efficacy, can show the guideline‐driven care. Further research into LS‐P‐NET associations, metastatic mechanisms, and therapy sequencing is essential for personalized management.

## 4 Discussion

This case represents a rare manifestation of Lynch syndrome (LS) that involves a metachronous non‐functional Grade 2 pancreatic neuroendocrine tumor (P‐NET) with hepatic metastases, it occurs 7 years after treatment of stage II colon adenocarcinoma. The patient harbors a pathogenic MLH1 variant (c.131C>A; p. Ser44Tyr), and developed this unusual malignancy at age 36, which underscores the broad clinical spectrum of LS that extends beyond traditionally recognized colorectal and endometrial cancers [4]. The constellation of molecular findings, particularly the intratumoral heterogeneity in MMR protein expression between primary and metastatic sites, illuminates the complex biological behavior of hereditary cancer syndromes.

LS patients face elevated pancreatic cancer risk, approximately 6.2% through age 80 for MLH1/MSH2/MSH6/PMS2 carriers versus 1.6% in the general population [13]. These malignancies typically manifest as ductal adenocarcinomas rather than neuroendocrine tumors [14]. Our literature review identified only three prior P‐NET cases in LS, none with metastatic disease. Barrera et al. described a 65‐year‐old woman with two incidental, non‐metastatic P‐NETs, one showing MLH1/PMS2 loss [14]. Sorscher et al. documented a high‐grade hepatic NET in a 63‐year‐old LS patient with MLH1 loss in both colon cancer and liver tumor [11]. Conversely, Karamurzin et al. reported a P‐NET with intact MMR expression, that leaves causality uncertain [11]. Our case uniquely presents the first documented intermediate‐grade, dMMR‐positive P‐NET with confirmed hepatic metastases in a young adult with LS.

The molecular characterization revealed findings of considerable biological interest. The primary pancreatic tumor (Grade 2, Ki‐67 4%–5%) demonstrated complete MLH1/PMS2 loss by immunohistochemistry, that is consistent with deficient mismatch repair [4]. However, hepatic metastases showed retained MLH1/PMS2 expression despite being neuroendocrine tumors with lower proliferative index (Grade 1, Ki‐67 1%). Next‐generation sequencing on tissue from both sites confirmed the germline MLH1 c.131C>A variant in both lesions, which establishes clonal relationship, while revealing discordant somatic mutational profiles with distinct genetic alterations at each site. No MLH1 reversion mutations were identified in metastatic lesions. This intratumoral heterogeneity, although uncommon, has been documented in LS‐associated colorectal cancers and multifocal tumors [11]. Several mechanisms may explain this pattern. Subclonal selection during tumor evolution could favor metastatic clones that regained or retained functional MMR expression through epigenetic modifications [8]. Variable “second hit” mechanisms may operate differently at primary versus metastatic sites. The primary tumor likely underwent somatic loss of the wild‐type MLH1 allele, while metastatic subclones may have retained it or developed compensatory changes [15] Additionally, the liver microenvironment may exert selective pressure favoring clones with intact MMR function due to differences in immune surveillance or metabolic stress [16].

These findings have a direct impact on treatment decision‐making. While immunotherapy with pembrolizumab has shown promise for dMMR/MSI‐H solid tumors [7], several considerations tempered enthusiasm for this approach in our patient. Well‐differentiated NETs characteristically have “cold” tumor microenvironments with limited inflammatory signaling [17].

Most critically, the metastatic disease, the actual therapeutic target, demonstrated intact MMR expression, potentially rendering it non‐responsive to checkpoint blockade [18].

Published literature on checkpoint inhibitors in dMMR NETs consists of isolated case reports with variable outcomes (Table 1). The multidisciplinary tumor board therefore elected not to pursue immunotherapy as frontline treatment [20] Instead, management followed consensus guidelines for neuroendocrine tumors [18]. The patient has initiated octreotide LAR 30 mg intramuscularly every 28 days, an approach justified by excellent performance status (ECOG 0), asymptomatic presentation, low tumor burden, and a low proliferative index—all predictors of favorable outcomes with somatostatin analog therapy.

**TABLE 1:** Lynch syndrome case reports with pancreatic neuroendocrine tumor.

Author, publication year	Case presentation	Family history of cancer	Genetic evaluation	Met criteria
Barrera et al. 2017 [19]	65 y/o F. At 45: screening colonoscopy: Lt. CRC (T1N0M0). At 57: Rt. CRC, Rt. Hemicolectomy. At 58: uterine Ca (T1N0M0): TAH + BSO. At 63: IDC of Rt. Breast. At 65: duodenal AC: PD+ PP: 2 accidental tumors (G1 7mm, ki67: 1%, G2 11mm, ki67:3%) in pancreas head (P‐NET). Recurrence free for 3 years.	1sth D: father: CRC, brother: gastric ca. MLH1 MMR gene mutation, c.731G > A (p. Gly244Asp) In all affected relatives.	Endometrial and CC: MLH1+, PMS2+ PNET: MLH1+, PMS2+, MSH2+, MSH6+ Other: MLH1‐, PMS2, MSH2+, MSH6+ ALL: MSI‐H	Both
Karamurzin et al. 2012 [11]	48 yo F. At 46: endometrial Ca: TAH. At 47: colon villous adenoma (G3), resected. At 48: abdominal pain: 2.2 cm mass (G1) in the pancreas body, resected. Pathology: P‐NET, IHC: chromogranin and synaptophysin.	1sth D: mother: CC and endometrial Ca 2nd D: 3 CC, 2 of them < 30, 1 endometrial Ca	Not reported	Bethesda
Our case	38 yo F. At 29: ascending colon adenocarcinoma (pT3N0): total colectomy. At 36: obstructive jaundice due to (pT2N0) PNET: Whipple procedure. 6 m later multiple liver lesions: metastasis from PNET.	1sth D: father: CRC, 2nd D: 3 of the patient's cousins: 2 CCs and 1 gastric Ca	CC: MLH1+, PMS2+ PNET: MLH1+, PMS2+ Liver lesion: INSM‐1+, MLH1+	Both

Abbreviations: AC, adenocarcinoma; BSO, bilateral salpingo‐oophorectomy; Ca, cancer; CC, colon cancer; CRC, colorectal cancer; cm, centimeter; G, tumor grade; IDC, invasive ductal carcinoma; IHC, immunohistochemistry; m, month, PD, pancreaticoduodenectomy; P‐NET, pancreatic neuroendocrine tumor; PP, pyloric preservation; Rt, right; TAH, total abdominal hysterectomy.

At three‐month follow‐up (June 2022), documented progression prompted transition to peptide receptor radionuclide therapy with Lu‐177 DOTATATE (Lutathera): 7.4 GBq intravenously every 8 weeks for four cycles, with octreotide LAR continuation between cycles. The patient has maintained durable disease control for over 3 years, remaining clinically well and symptom‐free. Furthermore, performing a Ga‐68 DOTATATE PET‐CT 6 months after resection—though not part of standard surveillance—proved diagnostically valuable, particularly given the patient's young age and underlying Lynch syndrome, which confer an elevated risk for metachronous malignancies [5]. With subtle hepatic changes on ultrasound, functional imaging successfully identified occult metastatic disease potentially less apparent on conventional imaging. Early detection of recurrence is critical in P‐NET management [21].

The comprehensive molecular profiling, including IHC for MMR proteins, MSI testing, MLH1 promoter methylation analysis, BRAF V600E testing, and NGS of both primary and metastatic lesions, reflects the level of care and attention that is essential when dealing with hereditary cancer syndromes [6, 12]. This approach distinguished germline from sporadic dMMRbn [8], confirmed the pathogenicity of the *MLH1* variant, established a clonal relationship despite discordant MMR expression, and provided a foundation for evidence‐based therapeutic decisions.

This case highlights several important considerations. First, expanding Lynch syndrome surveillance beyond colorectal and endometrial cancers may be warranted in high‐risk individuals [5, 13], though balancing the incremental benefit against the screening burden remains challenging. Second, establishing hereditary cancer registries with comprehensive reporting of unusual tumor presentations would facilitate genotype–phenotype correlations and evidence‐based screening recommendations [22]. Third, future research should include studies to understand clonal evolution and MMR heterogeneity, prospective trials evaluating therapy sequencing in LS‐associated NETs, and longitudinal studies defining true P‐NET incidence in LS populations [11, 13].

In conclusion, this case documents a novel presentation of Lynch syndrome featuring metastatic pancreatic neuroendocrine tumor with complex molecular characteristics, including intratumoral MMR heterogeneity. Successful management with guideline‐concordant therapies—somatostatin analogs followed by PRRT—resulted in sustained disease control for over 3 years, underscoring the importance of adherence to established treatment algorithms. This reinforces LS's expanding phenotypic spectrum [4, 5], the necessity for comprehensive molecular profiling [6], and the potential value of broadened surveillance strategies in hereditary cancer syndromes.

## Author Contributions

**Zahra Sadin:** data curation, formal analysis, investigation, project administration, supervision, validation, visualization, writing – original draft, writing – review and editing. **Fateme Salemi:** conceptualization, data curation, formal analysis, funding acquisition, investigation, methodology, project administration, resources, software, supervision, validation, visualization, writing – original draft, writing – review and editing. **Seyed Mohammad Reza Mortazavizadeh:** conceptualization, data curation, formal analysis, funding acquisition, investigation, methodology, project administration, resources, software, supervision, validation, visualization, writing – original draft, writing – review and editing. **Atefe Barzegari:** conceptualization, data curation, formal analysis, funding acquisition, investigation, methodology, project administration, resources, software, supervision, validation, visualization, writing – original draft, writing – review and editing. **Massih Bahar:** conceptualization, data curation, formal analysis, funding acquisition, investigation, methodology, project administration, resources, software, supervision, validation, visualization, writing – original draft, writing – review and editing. **Motahare Amiri:** conceptualization, data curation, formal analysis, funding acquisition, investigation, methodology, project administration, resources, software, supervision, validation, visualization, writing – original draft, writing – review and editing.

## Funding

The authors have nothing to report.

## Ethics Statement

Ethical approval was not required for this case report. However, all procedures performed were in accordance with institutional and national ethical standards.

## Consent

Written informed consent was obtained from the patient for publication of this case report and accompanying images.

## Conflicts of Interest

The authors declare no conflicts of interest.

## Data Availability

All data generated or analyzed during this study are included in this published article.
